# Acute macular neuroretinopathy as a manifestation of coronavirus disease 2019: A case report

**DOI:** 10.1002/ccr3.4976

**Published:** 2021-10-17

**Authors:** Marjan Masjedi, Mohsen Pourazizi, Nastaran‐Sadat Hosseini

**Affiliations:** ^1^ Isfahan Eye Research Center Isfahan University of Medical Sciences Isfahan Iran; ^2^ Isfahan Eye Research Center Department of Ophthalmology Isfahan University of Medical Sciences Isfahan Iran; ^3^ School of Medicine Isfahan University of Medical Sciences Isfahan Iran

**Keywords:** acute macular neuroretinopathy, COVID‐19, SARS‐CoV‐2, vasculopathy

## Abstract

The current findings is important in raising clinicians' awareness of the possibility of coincident acute macular neuroretinopathy (AMN) and COVID‐19 as a potential cause of retinal vascular damage and ischemia.

## Graphical abstract

The current findings are important in raising clinicians' awareness of the possibility of coincident acute macular neuroretinopathy (AMN) and COVID‐19 as a potential cause of retinal vascular damage and ischemia.

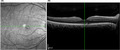

## 1 INTRODUCTION

This is a new case of simultaneous occurrences of AMN and COVID‐19 in a previously healthy woman. The importance of the current report lies in increasing the awareness of all physicians about the probable association of COVID‐19 with retinal vascular injury and ischemia. Acute macular neuroretinopathy (AMN) is a rare retinal involvement characterized by a wedge‐shaped reddish‐brown lesion extending toward the fovea, usually resulting in a sudden onset paracentral scotoma. The lesions are reported predominantly in young, healthy female individuals.^1^

The clear etiology of AVM is not entirely understood. Oral contraceptive use, caffeine, trauma, cocaine abuse, viral and flu‐like illness, sympathomimetic medications, and vaccination use are all factors associated with the AVM. Several studies have stated that most cases are attributed to an initial flu‐like condition, but no concrete evidence of a link with influenza has been identified to date.^2^

Exploring the literature elucidates the wide‐spectrum tissue tropism and multi‐organ complication of severe acute respiratory syndrome coronavirus‐2 (SARS‐CoV‐2) infection issued from the angiotensin‐converting enzyme 2 (ACE‐2), the primary receptor of the virus, since the coronavirus disease 2019 (COVID‐19) pandemic's onset. Ocular manifestations were initially described as ocular surface diseases such as conjunctivitis.^3^ Recently, there has been speculation about retinal involvement in fundus ophthalmoscopy and optical coherence tomography, including AVM.^4^, ^5^, ^6^ Virgo J et al. announced two cases of PAMM and AMN concurrent with the course of the SARS‐CoV‐2 infection.^7^

Our report describes a new instance of COVID‐19‐associated AMN that manifested as unilateral acute onset scotoma following SARS‐CoV‐2 infection in a 29‐year‐old woman who had no previous medical history to report. In addition to introducing this unique association, we review the relevant literature.

## 2 CASE PRESENTATION

A 29‐year‐old woman was presented with a chief complaint of experiencing acute onset paracentral visual field defect from 5 days ago in the left eye. She had a positive SARS‐CoV‐2 reverse transcription‐polymerase chain reaction (RT‐PCR) test in past medical history, performed following fever, headache, and non‐productive cough 2 weeks before the beginning of ocular symptoms. The symptom was mild, so the patient was advised to self‐quarantine without receiving any specific medication. The mild respiratory symptoms were relieved by the use of paracetamol and diphenhydramine. Previous ocular, medical and family histories were unremarkable except COVID‐19. She had no prior history of medications consumption, sun‐gazing, or eye trauma.

Ophthalmological examination revealed equally round and reactive to light and accommodation pupils with normal slit‐lam bio‐microscopy. Intraocular pressure was normal. The fundoscopic evaluation demonstrated a sharply contoured and well‐perfused disc as well as a yellow spot in the fundus left eye.

**FIGURE 1:**
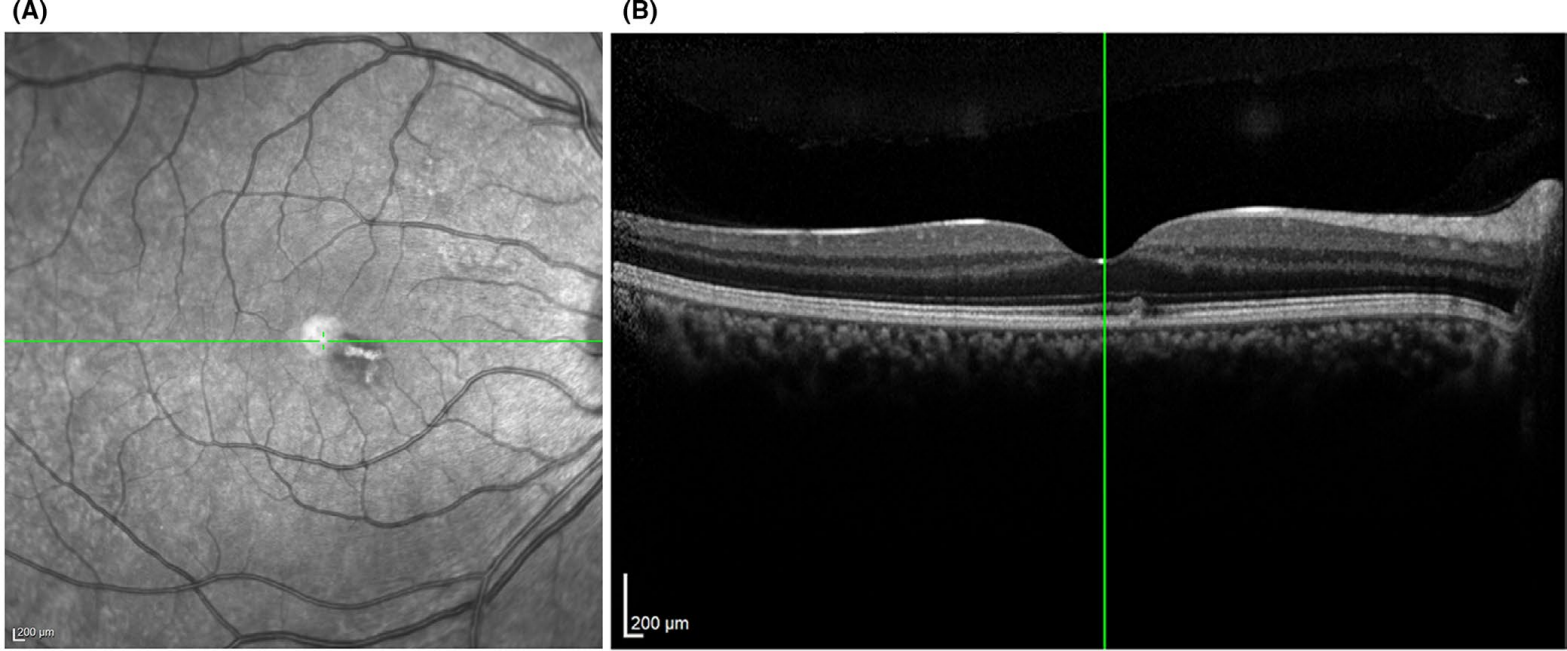
Acute macular neuroretinopathy associated with COVID‐19. (A) Infrared reflectance image displays wedged‐shaped lesion nasally to the fovea. (B) OCT demonstrated a focal area of hyper‐reflective change in the outer nuclear layer (ONL) and outer plexiform layer (OPL) associated with attenuation of the ellipsoid zone (EZ) nasal to the fovea of the left eye

Infrared imaging revealed a subtle, grayish wedge‐shaped lesion surrounding the fovea compatible with the hypo‐reflective area in the left eye. Additionally, spectral‐domain optical coherence tomography (SD‐OCT) demonstrated the disruption of the inner segment/outer segment junction (ellipsoid zone) (Figure 1). Based on patient history and ocular assessments, the possible diagnose of AMN associated with COVID‐19 was made.

**FIGURE 2:**
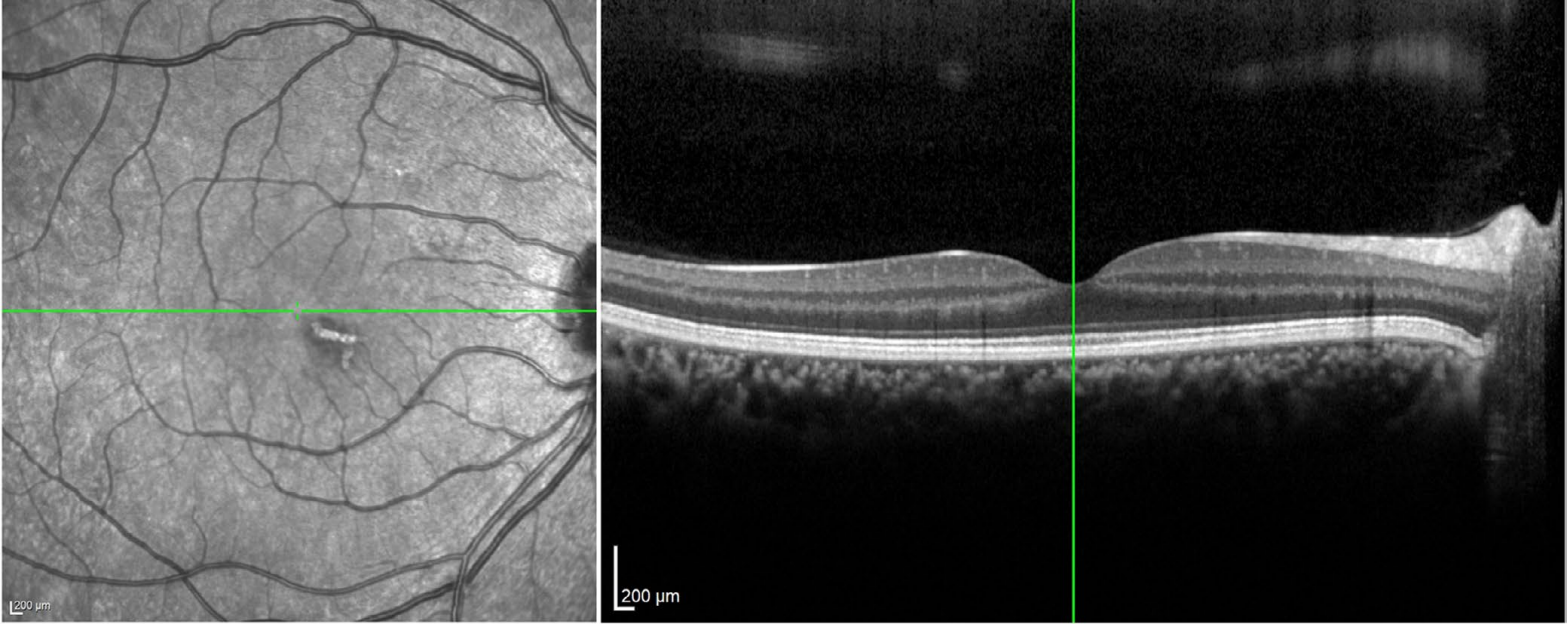
Acute macular neuroretinopathy associated with COVID‐19 after 2‐month follow‐up

Two months following the initial ophthalmology appointment, the condition began to fade gradually; however, the patient continued to complain of a visual field defect (Figure 2).

## 3 DISCUSSION

Although the exact etiopathogenesis of AMN is unclear, the proposed mechanism of AMN related to local or systemic vasculopathic injuries includes retinal vascular injuries or systemic underlying vasculopathic conditions.

The existence of ACE‐2 in the retina has indicated the posterior segment of the eye as a potential virus target. However, there is still a controversy on the verified relation between retinal damages and the SARS‐CoV‐2 infection. Discrepancies about the presence of SARS‐CoV‐2 RNA in the histopathologic analysis of the retina may warrant further investigations. Limited case reports highlighted that AMN could be related to SARS‐CoV‐2 infection.^4^, ^8^, ^9^

It was hypothesized that the retinal capillary plexus ischemia leading to AMN could be secondary to an acquired hypercoagulable state associated with COVID‐19, especially in the inflammatory phase. In the course of COVID‐19, on the one hand, hypercoagulable state results in pro‐thrombotic endothelial lesions and overactive immune response and cytokine storm, which cause local and systemic vascular injuries. On the other hand, the inactivation of ACE‐2 prompts an imbalance of the renin‐angiotensin system (RAS), leading to vasoconstriction.^4^, ^10^, ^11^ These pathological pathways result in retinal vasculature hypoperfusion, predisposing patients to ischemic retinal ischemia, including AMN.

Our case report features a convalescent COVID‐19 young female patient and a subsequent retinal involvement. SARS‐CoV‐2 infection is considered the presumptive etiology of retinal vascular ischemia without any other established etiological factors. There are clear limitations to the methodology of case studies in determining pathogenesis and predisposing factors, particularly in rare conditions. Nonetheless, we emphasize the potential association of AMN and COVID‐19. However, future researches are needed to understand the exact etiopathogenesis.

## CONFLICT OF INTEREST

There are no conflicts of interest reported by the authors. The authors are solely responsible for the article's content and writing.

## AUTHOR CONTRIBUTIONS

Marjan Masjedi involved in the patient management and performed investigation. Nastaran‐Sadat Hosseini wrote the initial draft and reviewed the literature. Mohsen Pourazizi revised the manuscript and approved the final version. All authors have contributed to the preparation of this manuscript and have reviewed it prior to submission.

## ETHICAL APPROVAL

Ethics approval for this report was obtained from the Ethics Committee of Isfahan University of Medical Sciences, Isfahan, Iran (IR.MUI.MED.REC.1400.423).

## CONSENT

The patient was provided with written informed consent to participate in the study.

## Data Availability

The data used to support this study's findings can be obtained from the corresponding author upon written request.
